# Dr. Alfred Bowlby

**Published:** 1916-01

**Authors:** 


**Dr. Alfred Bowlby,** of Waterford, Ont., died November 9, aged 96.

